# Red blood cell distribution width to albumin ratio and systemic immune-inflammatory index as predictors of mortality in severe pneumonia: A retrospective cohort analysis

**DOI:** 10.1371/journal.pone.0353695

**Published:** 2026-07-13

**Authors:** Lijia Shao, Rui Gong, Lihong Shen

**Affiliations:** Department of Clinical Laboratory, Affiliated Jinhua Hospital, Zhejiang University School of Medicine, Jinhua, Zhejiang, People’s Republic of China; Azienda Ospedaliero Universitaria Careggi, ITALY

## Abstract

**Objective:**

Severe pneumonia carries a high risk of mortality. There is a need for readily available prognostic biomarkers to improve risk stratification. This study evaluated the prognostic value of two novel composite indices, the red blood cell distribution width-to-albumin ratio (RAR) and the systemic immune-inflammation index (SII), in patients with severe pneumonia.

**Methods:**

This retrospective cohort study extracted data from electronic medical records of 194 adult patients (age ≥ 18 years) with severe pneumonia (diagnosed per Chinese guidelines) admitted to Jinhua Hospital (January 2022 to December 2024). After screening 2,268 admissions, 194 patients with complete 24-hour data were classified into survivors (n = 147) and non-survivors (n = 47). Associations between RAR, SII, and in-hospital mortality were analyzed using multivariate logistic regression, receiver operating characteristic (ROC) curves, and Kaplan-Meier survival analysis.

**Results:**

Compared with survivors, non-survivors had a significantly higher RAR (median: 0.44 vs. 0.37, *p* < 0.001) and SII (median: 2389 vs. 1870, *p* = 0.002). RAR was positively correlated with D-dimer (*r* = 0.254, *p* < 0.001), and negatively correlated with uric acid (UA) (*r* = −0.147, *p* = 0.042). SII was positively correlated with C-reactive protein (CRP) (*r* = 0.150, *p* = 0.037) and interleukin-6 (IL-6) (*r* = 0.188, *p* = 0.009). Advanced age (*OR*=1.042; 95%*CI*:1.004–1.082; *p* = 0.030), high RAR (*OR*=2.492; 95%*CI*:1.642–3.781; *p* < 0.001) and high SII (*OR*=1.575; 95%*CI*:1.074–2.309; *p* = 0.036) were independent risk factors for death in patients with severe pneumonia. The ROC of RAR and SII for predicting death in patients with severe pneumonia was 0.722 and 0.652, respectively. Patients with RAR < 0.43 had a higher cumulative survival than patients with RAR ≥ 0.43 (85.6% [119/139] vs. 50.9% [28/55]; *HR* = 4.987; 95%*CI*: 2.592–9.593; *p* < 0.001); Patients with SII < 2120 had a higher cumulative survival than patients with SII ≥ 2120 (86.9% [93/107] vs. 62.1% [54/87]; *HR* = 2.745; 95%*CI*: 1.531–4.924; *p* < 0.001).

**Conclusions:**

Elevated RAR and SII are significantly associated with an increased risk of in-hospital death in severe pneumonia patients. These indices may therefore serve as useful, readily available, and inexpensive prognostic tools.

## Introduction

Severe pneumonia remains a major respiratory disease associated with high morbidity and mortality [1]. The overall prevalence of severe pneumonia was 7.13 per 1000 person-years, in males 7.32 and females 6.93 per 1000 person-years, respectively, and varied markedly by region [2]. Although therapeutic and management approaches have progressed in recent years, challenges persist in achieving optimal patient outcomes [3]. Conventional markers such as interleukin-6 (IL-6), white blood cell count (WBC), and D-dimer are widely used, yet evidence regarding their prognostic value for mortality remains limited and controversial [4]. Given these limitations, there is a pressing need for more reliable prognostic tools. In recent years, combined inflammatory-nutritional indices have shown promise in critical illness prognostication.

Red blood cell distribution width (RDW), a conventional and readily available parameter in complete blood count analysis, reflects heterogeneity in red blood cell volume and has been increasingly linked not only to anemia but also to systemic inflammation and broader physiological dysregulation [5]. Elevated RDW may indicate the involvement of various pathological pathways, including inflammation, oxidative stress, telomere shortening, cellular aging, nutrient deficiencies, and impaired erythropoietin function [5–8]. For instance, Lippi et al. [6] demonstrated that RDW correlates strongly with inflammatory mediators such as C-reactive protein (CRP), IL-6, and TNF-α, while in critical illness, elevated RDW is also linked to oxidative stress, which disrupts erythrocyte membrane integrity. Furthermore, Kozlitina and Garcia [7] reported that higher RDW associates with telomere shortening and accelerated cellular aging, and nutritional deficiencies—particularly of iron, folate, and vitamin B12—as well as erythropoietin deficiency or hyporesponsiveness [8], contribute to increased RDW through impaired erythropoiesis. Although these mechanisms support the utility of RDW as a biomarker in severe pneumonia such as ventilator-associated pneumonia (VAP) and community-acquired pneumonia (CAP) [9–11], its combined use with other biomarkers remains underexplored.

Serum albumin (ALB), synthesized in the liver, is essential for maintaining colloid osmotic pressure and nutritional homeostasis [12]. ALB levels are influenced by chronic inflammation, liver function, and nutritional status [13]. Hypoalbuminemia is associated with malnutrition and increased mortality among hospitalized patients. The ratio of RDW to ALB (RAR) combines both inflammatory and nutritional dimensions [13], and has demonstrated prognostic value in conditions such as metabolic syndrome [14], cardiovascular disease [15,16], chronic kidney disease [17], and diabetes [18,19]. Nevertheless, its potential as a tool for risk stratification and clinical management in severe pneumonia is promising yet underexplored.

Alongside RAR, the Systemic Immune-Inflammation Index (SII)—calculated from neutrophil, platelet, and lymphocyte counts—has emerged as a significant inflammatory biomarker [20]. Initially identified in oncological research [21], SII has since demonstrated prognostic value across various cardiovascular conditions, including coronary artery disease, aortic stenosis, infective endocarditis, and heart failure [22–25]. More recently, its utility has been extended to respiratory infections. Studies indicate that SII serves as a useful prognostic tool for predicting the risk and severity of stroke-associated pneumonia (SAP) in patients with intracerebral hemorrhage (ICH), as well as the likelihood of ICU admission [26–29]. In the context of COVID-19, elevated SII levels have been associated with adverse outcomes, underscoring its potential role in severe pneumonia due to shared inflammatory pathways [30–33]. Despite these promising findings, the prognostic value of SII specifically in severe pneumonia has not yet been firmly established.

Given that RAR and SII capture complementary aspects of inflammation, nutrition, and immune response, and considering the limited evidence regarding their roles in severe pneumonia, this study aims to evaluate their prognostic utility for in-hospital mortality in this patient population. Through a retrospective cohort analysis, we seek to assess whether these indices can serve as practical tools for risk stratification and treatment guidance in individuals with severe pneumonia.

## Materials and methods

### Ethical approval and informed consent

This retrospective cohort study was conducted at Jinhua Hospital, Zhejiang University School of Medicine. The study protocol was reviewed and approved by the Institutional Ethics Committee of Affiliated Jinhua Hospital, Zhejiang University School of Medicine [(2025) Ethical Approval No. (106)]. The requirement for informed consent was waived due to the retrospective nature of the study and the use of fully anonymized clinical data. All procedures were performed in accordance with the ethical standards outlined in the Declaration of Helsinki.

### Study population and participant selection

**Fig 1:**
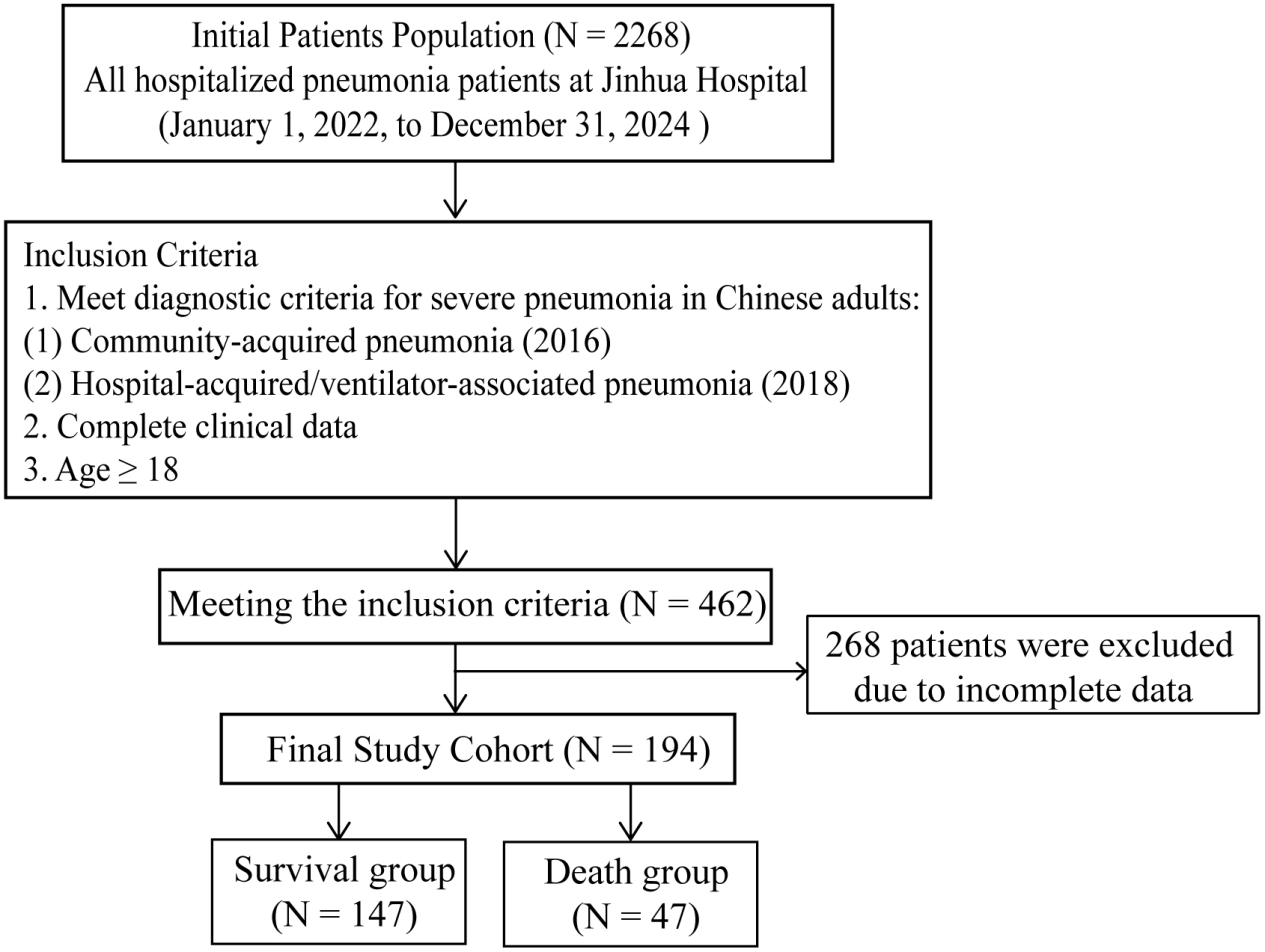
Study cohort flow diagram. The diagram illustrates the selection process of patients with severe pneumonia included in the analysis, starting from the initial hospitalized population (N = 2268) and concluding with the final study cohort (N = 194), which was divided into survival (N = 147) and death groups (N = 47).

We initially screened all adult patients (age ≥ 18 years) with a diagnosis of pneumonia who were admitted to our hospital between January 1, 2022, and December 31, 2024. The patient selection process is detailed in Fig 1. The diagnosis of severe pneumonia was established based on the criteria from the Chinese guidelines for community-acquired pneumonia (2016) [34] and hospital-acquired/ventilator-associated pneumonia (2018) [35]. Inclusion criteria were: (1) meeting the diagnostic criteria for severe pneumonia; (2) age ≥ 18 years; and (3) availability of complete clinical and laboratory data within the first 24 hours of admission. Exclusion criteria were: (1) incomplete clinical or laboratory data; and (2) not meeting the diagnostic criteria for severe pneumonia upon detailed review.

A total of 2268 patients were initially identified. After screening, 1806 patients were excluded for not meeting the inclusion criteria (primarily due to not meeting the diagnostic criteria for severe pneumonia or lacking laboratory confirmation). An additional 268 patients were excluded due to incomplete data. Consequently, 194 patients were enrolled in the final study cohort. Based on the primary outcome, these patients were categorized into two groups: the survival group (n = 147) and the death group (n = 47).

### Data collection and variables

Demographic, clinical, and laboratory data were retrospectively extracted from the hospital’s electronic medical record (EMR) system using a standardized data collection form. The data extraction and cleaning process was rigorously performed by trained researchers between April 17 and April 24, 2025. The following variables, collected within the first 24 hours of admission, were analyzed. Demographics and clinical characteristics: age, sex, body mass index (BMI), smoking status, and drinking history. Routine laboratory parameters: CRP, procalcitonin (PCT), IL-6, D-dimer, alanine aminotransferase (ALT), aspartate aminotransferase (AST), and uric acid (UA). Variables for key indices: RDW (%), ALB (g/dL), Platelet (PLT) count (×10⁹/L), neutrophil count (×10⁹/L), lymphocyte count (×10⁹/L). Calculated indices: RAR was calculated as: RAR = RDW (%)/ ALB (g/dL). SII was calculated as: SII = (PLT count × neutrophil count)/ lymphocyte count.

All laboratory measurements were performed in department of clinical laboratory using standardized automated analyzers: hematological parameters (RDW, PLT, neutrophil, and lymphocyte counts) were analyzed on a Sysmex XN-9000 automated hematology analyzer (Sysmex Corporation, Kobe, Japan) using the instrument’s standard impedance and flow cytometry methods. Biochemical parameters (ALB, ALT, AST, UA) were measured using standard enzymatic/colorimetric assays on a Beckman AU5800 clinical chemistry analyzer (Beckman Coulter, Brea, CA, USA); IL-6 and PCT were measured by fluorescence immunoassay on a Pylon 3D system (Pylon Iris, StarTorch Medical Systems, China); CRP was quantified using scattering immunoturbidimetry on a Jinrui PA 300 analyzer (Jinrui Biological Technology Co., Ltd., China).

### Study outcome

The primary outcome of this study was all-cause in-hospital mortality. Patients were followed from the date of admission until either hospital discharge or in-hospital death, with a maximum follow-up duration of 28 days. Subjects were categorized based on this outcome into the survival group and the death group for comparative analysis.

### Statistical analysis

Statistical analysis was performed using SPSS Statistics V22.0 (IBM Corp., Armonk, NY, USA). Continuous variables were assessed for normality using the Shapiro-Wilk test and presented as means ± SD for normally distributed data or medians (interquartile range, IQR) for non-normally distributed data. Categorical variables were expressed as frequencies (percentages). Student’s t-test or Mann-Whitney U test was used to compare continuous variables between groups, as appropriate, and χ²-test or Fisher’s exact test was used for categorical variables. Spearman correlation analysis was used to evaluate the relationship between RAR, SII, and other clinical indicators. Univariate binary logistic regression was first performed to identify potential risk factors for mortality. Variables with *p* < 0.10 in univariate analysis were included in the multivariate logistic regression model to identify independent risk factors for mortality, with results expressed as odds ratios (OR) and 95% confidence intervals (CI). Receiver operating characteristic (ROC) curve analysis was conducted to assess the predictive performance of RAR and SII for in-hospital mortality. Kaplan-Meier survival analysis with the log-rank test was used to compare cumulative survival rates between different RAR and SII groups, with optimal cut-off values determined by Youden’s index from ROC analysis. A two-tailed *p*-value < 0.05 was considered statistically significant.

## Results

### Baseline characteristics of study participants

A total of 194 patients with severe pneumonia were enrolled, including 147 in the survival group and 47 in the death group. As summarized in Table 1, significant differences were observed in several baseline and laboratory parameters between the two groups. Patients in the death group were significantly older than those in the survival group (73.8 ± 11.9 vs. 68.8 ± 11.6 years, *p* = 0.011) and had a higher prevalence of smoking history (51.1% vs. 34.0%, *p* = 0.036). Regarding inflammatory biomarkers, the death group exhibited markedly elevated levels of CRP (77.1 vs. 56.8 mg/L, *p* = 0.039), D-dimer (1125 vs. 820 mg/L, *p* = 0.021), and IL-6 (58.6 vs. 26.1 pg/mL, *p* = 0.003), along with significantly higher RDW (14.6% vs. 13.6%, p < 0.001) and PLT (282 vs. 240 × 10⁹/L, *p* = 0.005). Conversely, ALB and UA levels were significantly lower in the death group (ALB: 31.5 vs. 38.3 g/L, *p* < 0.001; UA: 251 vs. 274 μmol/L, *p* = 0.010).

**Table 1:** Demographic and characteristics of patients with severe pneumonia.

Characteristics	Survival group (*n* = 147)	Death group (*n* = 47)	*P* value
Age [years (means ± SD)]	68.8 ± 11.6	73.8 ± 11.9	0.011
Gender (male/female)	82/65	27/20	0.841
BMI [kg/m^2^ (means ± SD)]	22.2 ± 3.0	21.4 ± 3.0	0.137
Smoking habit [(*n*, %)]	50 (34.0)	24 (51.1)	0.036
Drinking habit [(*n*, %)]	23 (15.6)	7 (14.9)	0.901
CRP [mg/L (medians IQR)]	56.8 (25.1, 89.8)	77.1 (43.2, 121.9)	0.039
D-dimer [mg/L (medians IQR)]	820 (545, 1388)	1125 (598, 3561)	0.021
PCT [ng/mL (medians IQR)]	0.35 (0.15, 1.30)	0.45 (0.12, 1.32)	0.917
IL-6 [pg/mL (medians IQR)]	26.1 (10.6, 86.8)	58.6 (29.6, 146.1)	0.003
ALT [(U/L (medians IQR)]	46.0 (32.9, 63.3)	49.7 (41.8, 58.5)	0.286
AST [(U/L (medians IQR)]	47.1 (35.0, 63.6)	58.1 (43.4, 65.1)	0.057
UA [μmol/L (medians IQR)]	274 (218, 345)	251 (183, 303)	0.010
RDW [% (medians IQR)]	13.6 (13.0, 14.3)	14.6 (13.8, 15.1)	<0.001
ALB [g/L (medians IQR)]	38.3 (32.5, 42.5)	31.5 (26.5, 40.0)	<0.001
PLT [10^9^/L (medians IQR)]	240 (160, 312)	282 (208, 385)	0.005
Neutrophils [10^9^/L (medians IQR)]	8.40 (6.62, 9.84)	9.36 (6.92, 11.32)	0.035
Lymphocyte [10^9^/L (medians IQR)]	1.01 (0.79, 1.23)	1.05 (0.76, 1.23)	0.744

BMI, body mass index; CRP, C-reactive protein; PCT, procalcitonin; IL-6, interleukin-6; ALT, alanine aminotransferase; AST, aspartate aminotransferase; UA, uric acid; RDW, red blood cell distribution width; ALB, albumin; PLT, platelet; SD, standard deviation; IQR, interquartile range.

### Elevated RAR and SII in non-survivors

**Fig 2:**
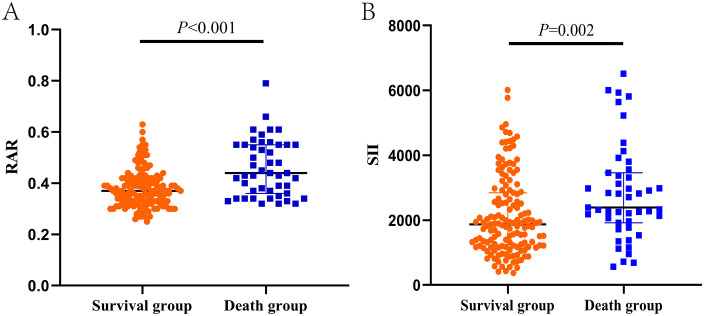
Distributions of RAR and SII in RDW/ALB and SII between the survival group and the death group. (A) RAR was significantly elevated in non-survivors compared to survivors (*p* < 0.001); (B) SII levels were significantly higher in non-survivors (*p* = 0.002). RAR, red blood cell distribution width to albumin ratio; SII, systemic immune-inflammatory index.

We further evaluated the prognostic utility of two composite indices: RAR and SII. RAR was significantly elevated in non-survivors (median: 0.44; IQR: 0.36–0.55) compared with survivors (median: 0.37; IQR: 0.33–0.42; *p* < 0.001) (Fig 2A). Similarly, SII was also higher in non-survivors (median: 2389; IQR: 1920–3460) than in survivors (median: 1870; IQR: 1202–2842; *p* = 0.002) (Fig 2B). These findings suggest that both indices may have utility for risk stratification in this patient population.

### Correlation of RAR and SII with established biomarkers

**Fig 3:**
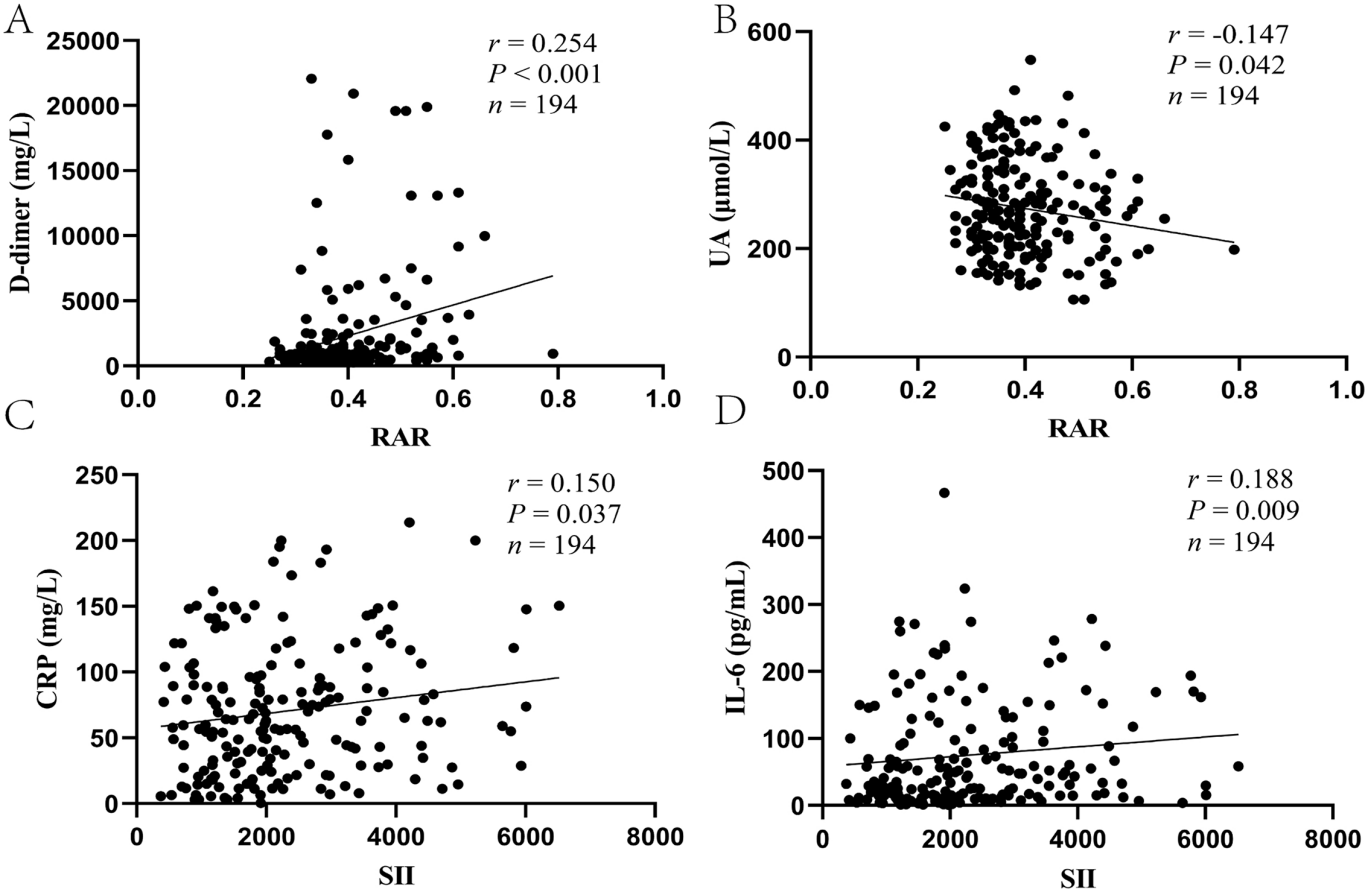
Correlation between RAR, SII and clinical indicators. (A) RAR was positively correlated with D-dimmer (*r* = 0.254, *p* < 0.001). (B) RAR was negatively correlated with UA (*r* = 0.254, *p* < 0.001). (C) SII was positively correlated with CRP (*r* = 0.254, *p* < 0.001). (D) SII was positively correlated with IL-6(*r* = 0.254, *p* < 0.001). RAR, red blood cell distribution width to albumin ratio; SII, systemic immune-inflammatory index; UA, uric acid; CRP, C-reactive protein; IL-6, interleukin-6.

To further explore the clinical relevance of RAR and SII, correlation analyses with established biomarkers were performed. Spearman correlation analysis revealed that RAR exhibited a significant positive correlation with D-dimer (*r* = 0.254, *p* < 0.001) (Fig 3A) and a significant negative correlation with UA (*r* = −0.147, *p* = 0.042) (Fig 3B). Conversely, SII showed significant positive correlations with both CRP (*r* = 0.150, *p* = 0.037) and IL-6 (*r* = 0.188, *p* = 0.009) (Fig 3C, 3D). These distinct correlation patterns indicate that RAR is more closely associated with pro-thrombotic and metabolic states, while SII more directly reflects the degree of systemic inflammation.

### RDW/ALB and SII as independent risk factors for mortality

Given the significant associations revealed by the univariate and correlation analyses, we next performed multivariate logistic regression to determine whether RAR and SII were independent predictors of mortality after adjusting for potential confounders.Variables with *p* < 0.10 in the univariate analysis were included in a multivariate logistic regression model to identify independent risk factors for mortality. The model was adjusted for smoking habit, CRP, D-dimer, IL-6, and UA.

As shown in Table 2, the analysis confirmed that after controlling for these confounding factors, both composite indices remained strongly and independently associated with an increased risk of in-hospital death. Specifically, for each standard deviation increase, a higher RAR was associated with an OR of 2.492 (95% *CI*: 1.642–3.781; *p* < 0.001), and a higher SII was associated with an OR of 1.575 (95% *CI*: 1.074–2.309; *p* = 0.036). Moreover, advanced age was also confirmed as an independent risk factor (*OR*: 1.042 per year; 95% *CI*: 1.004–1.082; *p* = 0.030).

**Table 2:** Multivariate logistic regression analysis of independent risk factors for in-hospital mortality in patients with severe pneumonia (n = 194).

Variable	*β*	*SE*	*Wald χ* ^*²*^	*P* value	*OR* (95% CI)
Age (per 1-year increase)	0.041	0.019	4.725	0.030	1.042 (1.004–1.082)
RAR (per 1-SD increase)	0.913	0.213	18.424	<0.001	2.492 (1.642–3.781)
SII (per 1-SD increase)	0.454	0.195	5.418	0.036	1.575 (1.074–2.309)

β, regression coefficient; SE, standard error; Wald χ², Wald chi-square statistic; OR, odds ratio; CI, confidence interval; SD, standard deviation; RAR, red blood cell distribution width to albumin ratio; SII, systemic immune-inflammatory index. The model was adjusted for smoking habit, CRP, D-dimer, IL-6, and uric acid.

### Superior diagnostic efficacy of RAR

**Fig 4:**
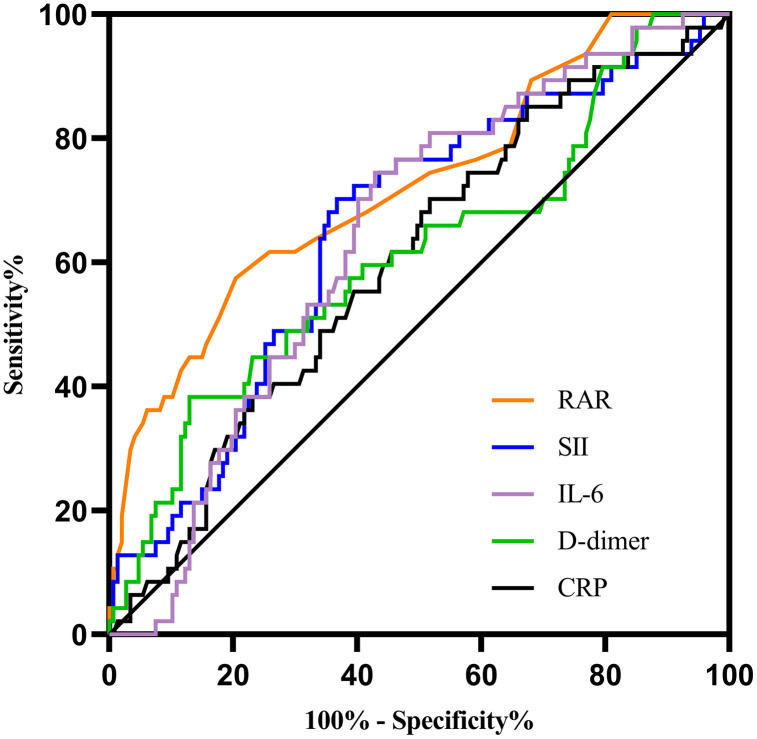
ROC curves of RAR, SII, IL-6, D-dimer and CRP for predicting in-hospital mortality. RAR, red blood cell distribution width to albumin ratio; SII, systemic immune-inflammatory index; IL-6, interleukin-6; CRP, C-reactive protein. The diagonal line represents the reference line (AUC = 0.5).

The predictive value of RAR, SII, CRP, D-dimer, and IL-6 for in-hospital mortality is shown in Table 3, and the corresponding ROC curves are presented in Fig 4. Among these indicators, RAR showed the highest diagnostic accuracy, with an AUC of 0.722 (95% *CI*: 0.635–0.808, *p* < 0.01). It had an optimal cutoff value of 0.43, providing a sensitivity of 57.4% and a specificity of 79.6%. SII had an AUC of 0.652 (95% *CI*: 0.563–0.741, *p* < 0.01), with an optimal cutoff value of 2120, achieving a sensitivity of 70.2% and a specificity of 63.3%. IL-6 demonstrated an AUC of 0.642 (95% *CI*: 0.559–0.726, *p* = 0.003), with an optimal cutoff value of 33.7 pg/mL, a sensitivity of 74.5%, and a specificity of 57.1%. D-dimer showed an AUC of 0.612 (95% *CI*: 0.516–0.709, *p* = 0.021), with an optimal cutoff value of 2351 mg/L, a sensitivity of 38.3%, and a specificity of 87.1%. CRP had an AUC of 0.600 (95% *CI*: 0.510–0.690, *p* = 0.039), with an optimal cutoff value of 55.5 mg/L, a sensitivity of 70.2%, and a specificity of 48.3%. Overall, RAR showed the best balance of sensitivity and specificity among the indicators evaluated.

**Table 3:** Diagnostic performance of various indicators for in-hospital mortality prediction (n = 194).

Variable	AUC	95% CI	*P* value	Optimal cutoff value	Sensitivity	Specificity
RAR	0.722	0.635–0.808	<0.01	0.43	57.4%	79.6%
SII	0.652	0.563–0.741	<0.01	2120	70.2%	63.3%
IL-6	0.642	0.559–0.726	0.003	33.7	74.5%	57.1%
D-dimer	0.612	0.516–0.709	0.021	2351	38.3%	87.1%
CRP	0.600	0.510–0.690	0.039	55.5	70.2%	48.3%

AUC, area under the curve; CI, confidence interval; RAR, red blood cell distribution width to albumin ratio; SII, systemic immune-inflammatory index; IL-6, interleukin-6; CRP, C-reactive protein. The AUC comparison with 0.5 was performed using the DeLong test. Optimal cutoff values were determined by the Youden index (maximum sensitivity + specificity – 1).

### Association of RAR and SII with survival

**Fig 5:**
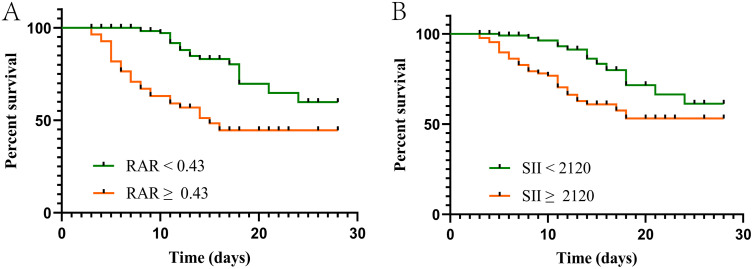
Kaplan Meier analysis of cumulative survival of severe pneumonia patients with different RAR and SII. (A) Patients with RAR < 0.43 exhibited significantly higher cumulative survival compared to those with RAR ≥ 0.43 (*p* < 0.001). (B) Patients with SII < 2120 had a significantly better survival probability than those with SII ≥ 2120 (*p* < 0.001). RAR, red blood cell distribution width to albumin ratio; SII, systemic immune-inflammatory index.

To further evaluate the prognostic potential of RAR and SII, Kaplan-Meier survival analysis was performed using optimal cutoff values derived from ROC analysis. Patients with RAR < 0.43 showed significantly better cumulative survival (85.6%) than those with RAR ≥ 0.43 (50.9%) (*HR* = 4.987; 95% *CI*: 2.592–9.593; *p* < 0.001) (Fig 5A). Similarly, patients with SII < 2120 exhibited improved survival outcomes (86.9%) compared to those with SII ≥ 2120 (62.1%) (*HR* = 2.745; 95% *CI*: 1.531–4.924; *p* < 0.001) (Fig 5B). These findings suggest that both RAR and SII may help stratify patients into distinct prognostic groups, with RAR appearing to have higher discriminatory power based on the observed hazard ratio.

## Discussion

Severe pneumonia remains a life-threatening condition with high mortality, and accurate prognostic assessment continues to pose significant challenges in clinical practice [1]. This study aimed to assess the prognostic value of RAR and SII in patients with severe pneumonia. Our findings indicated that both RAR and SII were significantly elevated in non-survivors and were independent predictors of in-hospital mortality.

Conventional single-parameter biomarkers remain widely used for managing infectious diseases. In our univariate analysis, these markers showed significant differences between survivors and non-survivors. D-dimer, CRP, and IL-6 exhibited modest predictive accuracy for mortality, with AUC values of 0.612, 0.600, and 0.642, respectively. Cataudella et al. [36] reported an AUC of 0.49 for CRP in elderly patients with CAP, which is somewhat lower than our estimate. Yu et al. [37] observed AUCs of 0.601 for D-dimer and 0.624 for CRP among elderly patients with severe pneumonia, while Wu et al. found that IL-6 predicted poor prognosis in COVID-19 with an AUC of 0.686 [38]—results showing closer alignment with our findings. A recent meta-analysis on COVID-19 also indicated higher prognostic accuracy for CRP (AUC = 0.84) and D-dimer (AUC = 0.69) [39]. Overall, these comparisons suggest considerable heterogeneity in the performance of single-parameter biomarkers, possibly due to variations in disease pathophysiology and cohort characteristics.

Furthermore, the loss of independent predictive value by single-parameter biomarkers in our multivariate analysis is consistent with the conclusion of Li et al. [40], which noted that individual biomarkers exhibit limited accuracy in predicting severe outcomes. Collectively, these results suggest that although conventional biomarkers may retain value for initial assessment and treatment monitoring, their utility as stand-alone tools for mortality risk stratification in heterogeneous conditions such as severe pneumonia is likely limited [41–43].

In contrast to single-parameter biomarkers, the composite indices RAR and SII demonstrated better prognostic performance in our study. RAR showed superior discriminatory power, with an AUC of 0.722 and higher specificity (79.6%), while SII exhibited higher sensitivity (70.2%). Moreover, both RAR and SII retained independent predictive value in multivariate analysis. For each standard deviation increase, a higher RAR was associated with an OR of 2.492 (95% *CI*: 1.642–3.781; *p* < 0.001), and a higher SII was associated with an OR of 1.575 (95% *CI*: 1.074–2.309; *p* = 0.036). The prognostic value of RAR is supported by several recent studies across different patient populations [44–46]. Chen et al. [44] reported that an ALB-RDW score independently predicted 90-day mortality in severe CAP with an AUC of 0.742, a result highly consistent with our finding. Similarly, Tan et al. [45] identified RAR as a robust predictor of outcomes among sepsis survivors (AUC = 0.790), and Hong et al. [46] found it predictive of mortality in diabetic foot ulcers. The consistency in these AUC values supports the reliability of RAR as a prognostic marker across various severe infectious and inflammatory conditions.

Furthermore, RAR demonstrated discriminative ability comparable to established clinical scoring systems such as CURB-65 (AUC 0.70–0.85) and PSI (AUC 0.76–0.87) [47–50]. Unlike these tools, which rely predominantly on static admission data and are limited by overreliance on age and categorical thresholds, RAR is derived from routine and dynamically measurable laboratory markers. This feature may allow for repeated risk assessment during hospitalization, capturing evolving inflammatory and nutritional states not fully reflected by conventional scores [50].

Beyond its prognostic utility, the RAR may reflect integrative pathophysiological processes in severe pneumonia. Non-survivors in our cohort exhibited significantly higher D-dimer levels, consistent with previous reports across various pneumonia subtypes [47,51,52], and a modest yet statistically significant positive correlation was observed between the RAR and D-dimer levels (*r* = 0.254, *p* < 0.001). This suggests a possible shared mechanism whereby proinflammatory cytokines may concurrently promote prothrombotic states (elevating D-dimer) [5], disrupt erythropoiesis (increasing RDW) [53], and suppress albumin synthesis [54]. Furthermore, non-survivors showed significantly lower serum UA levels, an important endogenous antioxidant, potentially indicating heightened oxidative stress—a finding aligning with the report by Inès Dufour et al. [55] in COVID-19, which described low serum UA as common and associated with disease severity and progression to respiratory failure requiring invasive mechanical ventilation. The modest inverse correlation between the RAR and UA (*r* = –0.147, *p* = 0.042) further supports the potential link between inflammatory-nutritional dysfunction and impaired antioxidant capacity. Thus, the RAR may serve as a composite indicator interlinked with inflammation, coagulopathy, oxidative stress, and malnutrition, all of which may collectively contribute to adverse outcomes in severe pneumonia [56–58].

Similarly, SII demonstrated independent prognostic value, albeit with lower discriminative ability than RAR (AUC 0.652). It significantly predicted mortality (*OR* = 1.575 per SD increase), with higher levels in non-survivors (median 2389 vs. 1870, *p* = 0.002). Positive correlations with CRP (*r* = 0.150, *p* = 0.037) and IL-6 (*r* = 0.188, *p* = 0.009) reinforce its role as a composite inflammatory marker. These findings align with reports in COVID-19 and other critical conditions where SII strongly predicted disease severity [59–61], underscoring its broad applicability.

In conclusion, our findings support RAR and SII as useful prognostic biomarkers in severe pneumonia. These composite indices outperformed conventional biomarkers and reflect integrative pathways involving inflammation, coagulation, oxidative stress, and nutritional status. Derived from routine, low-cost laboratory parameters, they can be readily incorporated into clinical workflows—particularly in resource-limited settings—and enable dynamic risk assessment through serial measurement. For example, a persistently elevated RAR may signal inflammatory and nutritional deterioration, prompting earlier intervention, while the combination of SII (high sensitivity) and RAR (high specificity) could improve risk stratification by ruling out low-risk and ruling in high-risk patients. Future studies should evaluate the efficacy of management protocols guided by these indices on clinical outcomes.

Our study has several limitations. Its single-center, retrospective design may limit generalizability and preclude causal inference, and residual confounding cannot be ruled out despite statistical adjustments. To address these limitations and translate our findings into clinical practice, we propose specific future research directions. First, a prospective, multicenter validation study is required to confirm the prognostic thresholds of RAR and SII and to develop a practical risk-stratification tool. Subsequently, interventional studies should evaluate whether dynamic monitoring of these indices can guide personalized therapy—such as intensified nutritional support or immunomodulation—and improve patient outcomes. Finally, integrating RAR and SII with established clinical scores such as CURB-65 to build a combined model may enhance predictive accuracy, potentially through advanced analytical approaches including machine learning.

## Conclusions

The composite indices RAR and SII may serve as integrative prognostic biomarkers in severe pneumonia, offering improved risk stratification and reflecting multiple pathophysiological pathways. Their clinical utility warrants further prospective validation.

## Supporting information

S1 Data The raw data. (XLSX)
